# Functional and cognitive impairment prevention through early physical activity for geriatric hospitalized patients: study protocol for a randomized controlled trial

**DOI:** 10.1186/s12877-015-0109-x

**Published:** 2015-09-15

**Authors:** Nicolás Martínez-Velilla, Alvaro Casas-Herrero, Fabrício Zambom-Ferraresi, Nacho Suárez, Javier Alonso-Renedo, Koldo Cambra Contín, Mikel López-Sáez de Asteasu, Nuria Fernandez Echeverria, María Gonzalo Lázaro, Mikel Izquierdo

**Affiliations:** Department of Geriatrics, Complejo Hospitalario de Navarra, Pamplona, Spain; Navarrabiomed-Fundación Miguel Servet, Pamplona, Spain; Department of Health Sciences, Public University of Navarra, Pamplona, Spain; Red de Investigación en Servicios Sanitarios en Enfermedades Crónicas (REDISSEC), Pamplona, Spain; IdiSNa. Navarra Institute for Health Research, Pamplona, Spain

## Abstract

**Background:**

Frail older adults have reduced functional and physiological reserves, rendering them more vulnerable to the effects of hospitalization, which frequently results in failure to recover from the pre-hospitalization functional loss, new disability or even continued functional decline. Alternative care models with an emphasis on multidisciplinary and continuing care units are currently being developed. Their main objective, other than the recovery of the condition that caused admission, is the prevention of functional decline. Many studies on functional decline have discussed the available evidence regarding the effectiveness of acute geriatric units. Despite the theoretical support for the idea that mobility improvement in the hospitalized patient carries multiple benefits, this idea has not been fully translated into clinical practice.

**Methods/design:**

This study is a randomized clinical trial conducted in the Department of Geriatrics of a tertiary public hospital with 35 beds allocated. Hospitalized patients who meet the inclusion criteria will be randomly assigned to the intervention or control group. The intervention will consist of a multicomponent exercise training programme, which will be composed of supervised progressive resistance exercise training, balance-training, and walking for 5–7 consecutive days. During the training period, patients will be trained in 20 min sessions twice a day (morning and evening).

**Discussion:**

Functional and cognitive impairment after and during acute hospitalization in older adults is a major determinant of the later need for health resources. If our hypothesis is correct and shows that a multicomponent, individualized and progressive exercise programme provides effective therapy for improving the functional capacity of acute elderly patients hospitalized for medical pathology versus conventional care, a change of the current system of hospitalization of elderly patients with medical conditions may be justified.

**Trial registration:**

ClinicalTrials.gov Identifier: NCT02300896 (Date of registration 19 November 2014)

## Background

Frail older adults have reduced functional and physiological reserves, rendering them more vulnerable to the effects of hospitalization, which frequently results in failure to recover from the pre-hospitalization functional loss [1], new disability [2], or even continued functional decline [3]. Furthermore, consequences arise at multiple levels including cognitive impairment, longer hospital stays and institutionalization, poor mood, delirium, deconditioning, aspirations, pressure ulcers, falls, decreased caloric intake, social isolation, poor quality of life, increased use of health-related resources, disability and death [3–16].

Traditional risk factors for functional decline secondary to hospitalization are usually associated with comorbidities, malnutrition, depression, age, severity of illness and cognitive status [17–19]. However, the current model of care for hospitalized older adults plays an important role as a risk factor for in-hospital functional deterioration and has only recently begun to be evaluated [4, 20]. In-hospital mobility seems to be directly related to post-hospitalization functional outcomes [4, 20] and is one of the strongest predictors of functional decline. Hospitalized elderly patients are often bedridden; some studies show that more than 83 % of these patients are bedridden versus 4 % who are permitted to stand or walk [21, 22]. In older adults hospitalized for nondisabling conditions, in-hospital risk factors such as low mobility account for immediate and 1-month post-hospitalization functional declines [23]. Furthermore, illnesses and injuries that lead to hospitalization increase the likelihood of transitioning from non-frail to pre-frail, frail, or greater frailty states. Moreover, increasing evidence has shown that many older individuals have the capacity to recover from frailty and pre-frailty, although the likelihood of attaining a less frail state is lower. This probability can be reduced by approximately 50 % for each intervening hospitalization [24]. The figures regarding functional decline during hospital admission are heterogeneous and vary from 38-80 % depending on the study [10, 20, 25–27]. In a study conducted in our department [28], secondary functional impairment on admission was noted in 80 % of the patients susceptible to such impairment, persisting at discharge in 30 % of the patients.

Muscle strength and aerobic capacity decrease rapidly as a result of immobilization. After only ten days of rest, a healthy elderly person can lose 12–14 % of their VO_2max_ and muscle strength in the lower extremities [29]. In addition, skeletal muscle power decreases more rapidly than muscle strength with advancing age [30] and is also strongly associated with functional outcomes and functional capacity in elderly individuals at risk of disability [31, 32]. At the muscular level, reduced muscle use is associated with myofibrillar protein loss, muscle atrophy, and impaired control of the recruitment of motoneurons; at the clinical level, reduced muscle use is associated with decreased coordination, muscle strength, power output, aerobic capacity, balance, and exercise tolerance [5]. The consequences usually extend over time, and may produce long-term effects [30].

Alternative care models for an emphasis on multidisciplinary and continuing care units are currently being developed. Their main objective, other than the recovery of the condition that caused admission [32], is the prevention of functional decline. Many studies on functional decline have discussed the available evidence regarding the effectiveness of acute geriatric units [33, 34]. Despite the theoretical support for the idea that mobility improvement in the hospitalized patient carries multiple benefits, this idea has not been fully translated into clinical practice, and some studies have found paradoxical results [35]. The new models include exercise as an essential part of conventional treatment, at least when the patients are discharged to their homes [36]. Simple and basic procedures such as increasing the walking duration by 12 min or daily slow walking can reduce the average hospital stay [37]. In all of these circumstances, a comprehensive geriatric assessment of this type of patient should also consider the close link between the functional and cognitive situations, in addition to the previous theoretical concepts [38].

Exercise and early rehabilitation programmes are among the mechanisms by which functional and cognitive decline is prevented during hospitalization. Although risk factors associated with hospitalization and functional decline after discharge have been intensively studied, few randomized clinical trials have examined the potential benefits of conducting standard exercise programmes for hospitalized acute elderly medical patients. Nevertheless, the theoretical framework allows us to grasp the scope of possible improvement that exists for this population sector when such interventions are applied properly and selectively. The benefits of exercise have been clinically, biologically and even economically confirmed [39, 40], making exercise part of the therapeutic arsenal at our disposal. Multicomponent programs, and especially resistance exercise that includes muscle power training, are currently the most relevant interventions to slow down disability and other adverse outcomes, but these programmes have not been tested in acute geriatric patients. Moreover, to be effective, exercise has to be prescribed with a progressive individualized plan, similar to other medical treatments [31]. Some prospective studies have previously shown that hospitalization of older adults in a suitable environment can reduce disability and enhance the recovery of compromised activities during and after the acute event, which is contrary to some theories that highlight only the negative aspects or removal from the living environment [41].

The Cochrane reviews regarding exercise for acutely hospitalized elderly medical patients included only seven randomized controlled trials and two controlled clinical trials out of 3138 potentially relevant articles; the effect of exercise on measures of functional outcome was uncertain, and no effects of intervention on adverse events were found. A small reduction in the stay and total hospital costs (silver-level evidence) was found [42]. However, very few studies have explored the feasibility of conducting exercise programmes for hospitalized acute elderly patients [43]. Furthermore, evidence is lacking to determine which types of hospitalized elderly patients would benefit more from each programme and whether each programme is viable.

## Study design and setting

**Fig. 1:**
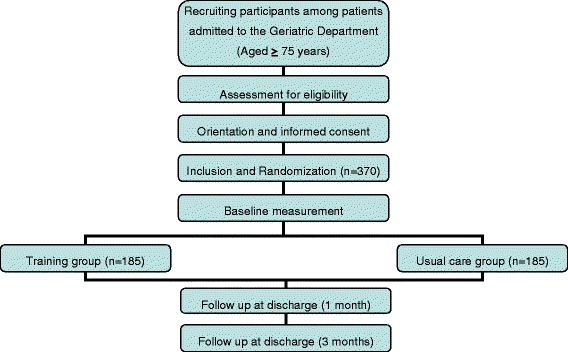
Flow diagram of the study protocol

This study is a randomized clinical trial conducted in the Department of Geriatrics of a tertiary public hospital with 35 beds allocated. Hospitalized patients who meet the inclusion criteria will be randomly assigned to the intervention or control group. Patient recruitment will begin within the first 48 h of admission to the ward, and these patients will be identified through a list of patients admitted to the hospital and assigned to the Department of Geriatrics. The study flow diagram is shown in Fig. 1. After signing an informed consent form, the subjects will be randomly assigned (as explained below) to either the intervention or control group. The researcher who decides whether the patient is assigned to the intervention or control group will not be the attending physician. Patients or their relatives (if the patient has cognitive impairment) will be informed of the random inclusion in one group, but will not be informed as to which they belong. The data for both the intervention group and the control group will be obtained at four different times: the initial visit during the acute hospitalization, at discharge, and at one and three months after discharge from the outpatient clinic. Time of measurement of the different variables is shown in Table 1. The protocol employs relevant standard protocol items for clinical trials according to the SPIRIT 2013 statement [44] and follows the CONSORT statement [45] for transparent reporting. The trial is registered at ClinicalTrials.gov, identifier NCT02300896.

**Table 1:** Time of measurement of the different variables on the participants of the study

Measurement	T1 Baseline	T2 After training or control period	T3 1-month	T4 3-months
Categorical scale of pain	X	X	X	X
Barthel Index	X	X	X	X
Geriatric depression Scale of Yasavage	X	X	X	X
Mini-Mental State Examination (MMSE)	X	X	X	X
Short Physical Performance Battery (SPPB)	X	X	X	X
Gait Velocity Test (GVT)	X	X	X	X
Dual-task (verbal and counting GVT)	X	X	X	X
Maximal isometric force of handgrip, knee extension and hip flexion	X	X	X	X
1RM (Leg press, Chest press and Knee extension)	X	X	X	X
Muscle power at 50 % 1RM in Leg press	X	X	X	X
Confusion Assessment Method (CAM)	X	X	X	X
Quality of Life (EQ-5D)	X	X	X	X
Geriatrics syndromes	X	X	X	X
Isaacs set test	X	X	X	X
Trail Making Test (TMT)	X	X	X	X
Laboratory parameters	X			
Diseases considered grouped by ACG of Salisbury and CIE-10 codes	X			
Cumulative Illness Rating Scale for Geriatrics (CIRS-G)	X			
Zarit Scale	X			
Falls	X	X	X	X
Mini Nutritional Assessment (MNA)	X			

Adverse events, including muscle pain, fatigue, and general aches and pains will be recorded by the training and testing staff and by self-report during the study period. We will also record the number of falls during the study and for one year prior to admission.

This study has been approved by the Navarra Clinical Research Ethics Committee (Pyto 23/2014).

### Study participants and eligibility criteria

Individuals over 75 years of age admitted to the Department of Geriatrics of the Complejo Hospitalario de Navarra between March 2015 and March 2017.

The inclusion criteria are:

- Age: 75 years or older.
- Able to ambulate with or without personal/technical assistance.
- Barthel Index ≥60
- Able to communicate.
- Informed consent: Must be capable and willing to provide consent.

The exclusion criteria are:

–Duration of hospitalization < 6 days.
–Any factor precluding performance of the physical training programme or testing procedures as determined by the attending physician. These factors include, but are not limited to the following:
  - Terminal illness.
  - Myocardial infarction in the past 3 months.
  - Unstable cardiovascular disease or other medical condition.
  - Upper or lower extremity fracture in the past 3 months.
  - Severe dementia (GDS 7).
  - Unwillingness to either complete the study requirements or to be randomized into the control or intervention group.

### Randomization and blinding

The study participants will be randomized (www.randomizer.org) into an intervention group and a control group. Participants will be explicitly informed and reminded not to discuss their randomization assignment with the assessment staff. The assessment staff will be blinded to the participant randomization assignment, as well as to the main study design and to what changes we expect to occur in the study outcomes in either group.

It will not be possible to conceal the group assignment from the staff involved in the training of the intervention group.

Patients or their families (if the patient has cognitive impairment) will be informed of the random inclusion in one group, but will not be informed as to which group they belong.

### Statistics and sample size

The required simple size to detect a difference of 15 % in the frequency of patients that get at discharge a functional improvement greater than 10 points in Barthel Index is 161 patients in each group. Assuming a loss of 15 % of patients in the follow-up, we fixed a final sample size of 185 patients per group.

In an initial descriptive analysis, for qualitative variables we will calculate frequencies and confidence intervals, and for continuous variables, statistics of central tendency and dispersion such as means, standard error and confidence intervals or median and interquartile range. In order to assess the extent of the therapeutic effect, we will compute for every patient the difference between final and initial level of the outcome variables. Normality of continuous variables will be checked graphically and through K-M and Shapiro-Wilk tests, and their differences between groups by means of parametric tests (T-Tests, ANOVA) or non-parametric tests (Mann–Whitney U, Kruskal- Wallis). A Bonferroni post-hoc test will be used to evaluate statically significant (*p* < 0.05) group and time differences. Associations between clinical and biomechanical tests will be reported by their correlation coefficient (r value), level of significance (p value), and the amount of variance explained (r2 value). Values of r will be used to indicate small (r = 0.10), medium (r = 0.30), and large (r = 0.50) size correlations (i.e., effect size). Finally, the relationship between qualitative variables will be assessed through χ^2^ and Fisher exact tests. The level of statistical significance will be 0.05. Data will be analyzed with SPSS package 21.0

## Detailed description

### Usual care group (control)

Participants randomly assigned to the usual care group will receive normal hospital care, which includes physical rehabilitation when needed.

### Intervention group (training)

The intervention will consist of a multicomponent exercise training programme [46], which will be composed of supervised progressive resistance exercise training, balance-training, and walking for 5–7 consecutive days. During the training period, patients will be trained in 20 min sessions twice a day (morning and evening).

The supervised multicomponent exercise training programme will be comprised of upper and lower body strengthening exercises, tailored to the individual’s functional capacity, using weight machines and aiming for 2–3 sets of 8–10 repetitions at an intensity of 30–60 % of 1RM (Matrix, Johnson Health Tech, Ibérica, S.L. Torrejón de Ardoz, Madrid, Spain) combined with balance and gait retraining exercises that progressed in difficulty and functional exercises, such as rises from a chair. The second part of the session will consist of functional exercises such as knee extension and flexion, hip abduction, balance movements, and daily walking in the hospital. A minimum of 2 days elapsed between consecutive training sessions. The resistance exercises focused on the major upper and lower limb muscles. Each resistance training session will include 2 exercises for the leg extensor muscles (bilateral leg extension and bilateral knee extension muscles) and 1 exercise for upper limbs (seated bench press). During the progressive resistance training, instruction will be provided to the participants to perform the exercises at a high velocity of motion. However, care will be taken to ensure that exercises were executed with correct form. In each session, subjects will perform a specific warm-up with one set of very light loads for the upper and lower body. Balance and gait retraining exercises that progressed in difficulty will be also implemented: semi-tandem foot standing, line walking, stepping practice, walking with small obstacles, proprioceptive exercises on unstable surfaces (foam pads sequence), and altering the base of support and weight transfer from one leg to the other. One experienced physical trainer will carefully supervise all training sessions. The training sessions will last for approximately 40 min. The approximate duration of each part of the training will be: 5 min of warm-up, 10 min balance and gait retraining, 15 min of resistance training, and five minutes of stretching (cool-down). The training protocol is shown in Table 2. Participants and their family members will be carefully familiarized with the training procedures in advance.

**Table 2:** Intervention group exercises

	Exercise	Day 1	Day 2	Day 3	Day 4	Day 5	Day 6*	Day 7*
Morning	Rises from a chair	1×5	1×10	2×10	3×10	3×8	3×8	3×8
	Leg press	1RM + 1×10 (30 % 1RM)	2×10 (30 % 1RM)	3×10 (40 % 1RM)	3×10 (50 % 1RM)	3×8 (60 % 1RM)	3×8 (60 % 1RM)	3×8 (60 % 1RM)
	Chet press	1RM + 1×10 (30 % 1RM)	2×10 (30 % 1RM)	3×10 (40 % 1RM)	3×10 (50 % 1RM)	3×8 (60 % 1RM)	3×8 (60 % 1RM)	3×8 (60 % 1RM)
	Leg extension	1RM + 1×10 (30 % 1RM)	2×10 (30 % 1RM)	3×10 (40 % 1RM)	3×10 (50 % 1RM)	3×8 (60 % 1RM)	3×8 (60 % 1RM)	3×8 (60 % 1RM)
Afternoon	Leg extension (0,5 – 1,0 Kg)		2×10	2×10	2×10	2×10	2×10	2×10
	Leg flexion (0,5 – 1,0 Kg)		2×10	2×10	2×10	2×10	2×10	2×10
	Hip abduction (0,5 – 1,0 Kg)		2×10	2×10	2×10	2×10	2×10	2×10
	Hand grip ball		2×10	2×10	2×10	2×10	2×10	2×10

*In case that the patient is still hospitalized

### Outcome measures

#### Primary outcome

The primary outcome measure is the change in functional and cognitive status during the study period. The functional capacity of patients will be evaluated by the Short Physical Performance Battery (SPPB) [47], which evaluates, balance, gait ability, and leg strength using a single tool. The total score will range from 0 (worst) to 12 points (best). The SSPB test has been shown to be a valid instrument for screening frailty and predicting disability, institutionalization, and mortality. A total score of less than 10 indicates frailty and a high risk of disability and falls. One-point change in the score has clinical relevance [48, 49]. Loss of handgrip of the dominant hand is a useful tool for the measurement of functional capacity. It is a strong predictor of disability, morbidity, and mortality as well as one of the components of Fried’s frail phenotype. Furthermore, the functional status of patients will also be assessed before measurements with the Barthel Index, an international and validated tool of disability. The scores range from 0 (severe functional dependence) to 100 (functional independence) [50]. Gait ability will be assessed using the 6-metre gait velocity test (GVT). Starting and ending limits will be marked on the floor with tapelines for a total distance of 8 m. Participants will be instructed to walk in their self-selected usual pace for two attempts. The results of both trials will be averaged to obtain a single value. The first and last metre, considered the warm-up and the deceleration phases, respectively, will not be included in the calculations of the gait assessment. Dual task conditions (gait evaluation during the simultaneous performance of a cognitive motor action) have recently been recognized as a sensitive assessment method for interactions between cognition, gait, falls and frailty. Changes of gait parameters (i.e., gait velocity and gait variability) while performing a dual task test (dual task cost) could be early predictors of falls risk (50) and useful tools for functional evaluations in frail elderly patients. Exercise can modify dual task cost and consequently fall risk and functional capacity (31). The dual-task paradigm [51] will be used in the 6-m habitual gait velocity test (GVT). Two trials will be performed to assess the gait velocity while performing a verbal or counting task (verbal GVT and counting GVT, respectively). During the verbal dual-task condition (verbal GVT), we will measure the gait velocity while participants are naming animals aloud. During the arithmetic dual-task condition (counting GVT), we will assess the gait velocity while participants are counting backward aloud from 100 by ones. The cognitive score will be measured by counting the number of animals named (dual-task with verbal performance) or determining how many numbers were counted backward (dual-task with arithmetic performance). Isometric upper (right hand grip) and lower limb (right knee extensors and hip flexors) muscle strength will be measured using a manual dynamometer. Maximal dynamic strength will be assessed using the 1RM test in the bilateral leg press, knee extension and bench press exercises using exercise machines (Exercycle, S.L., BH Group, Vitoria, Spain). In the first assessment, the subjects will warm up with specific movements for the exercise test. Each subject's maximal load will be determined in no more than five attempts, with a 3-min recovery period between attempts. After determination of the 1RM values, the subjects will perform ten repetitions at maximal velocity at intensities of 50 % of 1RM to determine the maximum power (w) and the loss of power during the ten repetitions in the leg press machine. The power will be recorded by connecting a velocity transducer to the weight plates (T-Force System, Ergotech, Murcia, Spain). During all neuromuscular performance tests, a strong verbal encouragement will be given to each subject to motivate them to perform each test action as optimally and rapidly as possible. Qualified fitness specialists will individually monitor and carefully supervise all training sessions and provide instruction and encouragement during all sessions. Distribution of the training sessions throughout the day should minimize cumulative fatigue and help to maintain adherence. Adherence to the exercise intervention programme will be documented in a daily register of sessions. Changes in cognitive-affective status after the intervention will be measured using the Mini Mental State Examination, Yesavage GDS and Trail Making Test (Table 3).

**Table 3:** Collected variables

1. Baseline measurements: Outcomes measures will be collected on the test day written in an information sheet.	
1.1. Individual characteristics:	
Demographic variables	Information regarding the age and the gender of the patients will be collected.
Functional status	Reflects the ability of the patient for performing activities of daily living, as well as the capacity to relate with others and participating in society. It will be measured with the Barthel Index.
Functional capacity	SPPB, Gait velocity, Handgrip, dual tests.
Cognitive function	Highlights cognitive impairments that might interfere with self-care and independence in elderly patients. In the present study, we will use the Mini Mental State Examination, and the Trail Making Test as executive function parameters, as well as the Confusion Assessment method for delirium evaluation, and the Geriatric depression Scale of Yesavage as an indicator of psychosocial status.
Caregiver burden	Will be measured through Zarit scale.
Nutritional status	Indicates malnutrition risk in elderly patients. In addition to the weight and height data, information related to factors that increase the risk of malnutrition will be collected. These will be measured via MNA test.
Quality of Life	Evaluates the individual’s social well being, due to its easiness in administration, validity and reliability, the EuroQol-5D is one of the questionnaires with largest diffusion and validity.
Geriatrics syndromes	Characterised by the simultaneous presence of illnesses, clinical and functional conditions that can usually lead to incapacity. The specific presence of immobility, incontinence, constipation, pressure ulcers, cognitive impairment, delirium, depressive tendencies, falls, insomnia, visual impairments, hearing impairments, malnutrition, dysphagia, and pain.
Comorbidity	Will be measured by means of Cumulative Illness Rating Scale-Geriatrics (CIRS-G).
1.2. Intervention-measurements	
Upper and lower strength	Maximal isometric force of knee extension, handgrip and hip flexion.
Dynamic muscle power on variable resistance exercise machine.	Will be measured through a T-force system device, connected to the variable resistance machine, so it is able to assess the velocity and power of every single lift.
Kinematic variables of human movement.	Gait patterns of the patients will be recorded by a triaxial accelerometer while performing the GVT. This small device traces acceleration force, speed and angular position data in the three planes.
2. Follow-up: Institutionalization, survival, functional impairment, quality of life, health care resources use (e.g. GP visits emergencies, hospital admission, medicine consumption).	

Secondary Outcome Measures:

- Quality of life: EuroQol Scale
- Delirium: Confusion Assessment Method
- Mortality: Number of days alive after admission to the hospital
- Use of health resources: New admissions to the hospital, admission to nursing homes, and visits to the general practitioner
- Falls

## Discussion

Functional and cognitive impairment after and during acute hospitalization in older adults is a major determinant of the later need for health resources. If our hypothesis is correct and shows that a multicomponent, individualized and progressive exercise programme provides effective therapy for improving the functional capacity of acute elderly patients hospitalized for medical pathology versus conventional care, a change of the current system of hospitalization of elderly patients with medical conditions may be justified. While the current system does not promote the execution of a scheduled exercise routine during the hospitalization period, if we can modify the current guidelines, it is likely that patients will present lower levels of functional and cognitive impairment after the hospitalization period, experience a better quality of life, produce lower consumption of healthcare resources (less readmissions and lower institutionalization), and finally, exhibit reduced mortality.

This trial is also relevant because exercise interventions in elderly patients have usually been performed in participants in the community, institutions or hospitalized for rehabilitation purposes. Frequently, older patients with multiple comorbidities are routinely excluded due to acute medical conditions. To date, few randomized clinical trials have been conducted and normally these trials use heterogeneous interventions (sometimes poorly explained), while our study allows the extrapolation of results through a well-defined methodology applied to other areas. The introduction of an exercise programme in hospitalized elderly patients as well as being viable and likely producing no increase in costs, could have a significant impact on both the short and long term by improving health care and functional parameters. Moreover, if our results are as expected, a possible new targeted and therapeutic tool during hospitalization for these complex patients could be developed and implemented in hospitals everywhere. We believe that, as with other medical treatments, the programme should be planned, individualized and monitored.

Another innovative aspect of our study compared with the few clinical trials published so far is the utilization of an interdisciplinary team that manages not only the clinical aspects but also the physiotherapy and engineering kinematics. Furthermore, in the case that the means used for experimental quantification of the power and muscle strength kinematic variables are feasible, it raises the possibility of incorporating commonly used means and patenting both the systematic interventions and the mechanisms of quantification

## Trial status

The trial commenced recruitment on March 5, 2015 and is currently open for recruitment. Recruitment will cease when 370 participants have been randomized. It is anticipated this target will be reached by March 2017.
